# Epistle to the Delinquents

**Published:** 1910-03

**Authors:** 


Epistle to the Delinquents.—A true physician is essentially a gentleman. A gentleman has a high sense of honor. He discharges all obligations, and pecuniary obligations are essentially matters of honor. It is hard to understand how any reputable physician can reconcile it to his self-respect and the crudest sense of honor to take, read and enjoy a publication month after month, year after year, for which he has subscribed and never countermanded, and utterly ignore every appeal made to him for settlement. And it is almost incredible that there are physicians of high standing who will ignore a subscription bill of a few dollars, sent him four times a year for two, three, four, five or six years. Attention has been called time and again to the ruling of the Post-office Department requiring publishers to drop all such delinquents. But still there are many from whom we have not heard. Surely no self-respecting physician whose subscription is in arrears will allow us to be forced to cut him off, so long as he is in arrears. It is a matter of honor. We have no discretion. We need the money, and want the friendship and support of these men, and trust this last appeal will bring a prompt response. * * * The season of "good cheer" and good will toward man has just passed. The subscription you owe us would be gratefully received. It would cheer us onward, and make you feel that you had discharged a duty and help us in doing likewise.

We beg to assure you that your promptness is appreciated. We have reason to be encouraged at the constant stream of letters of appreciation with checks and postoffice money orders which have been pouring in since the middle of December, when bills for subscription were rendered to every name on our subscription books. The aggregate due on subscriptions from all sources runs well up into the thousands. Every doctor who received a statement can very easily afford to send us the amount. It is a small amount to him, but the aggregate of these small bills will go a great ways towards paying paper and printing bills. To those of you who have not yet responded, we beg to advise you that we expect you to do so at an early date. Members of the honorable profession of medicine should not neglect to remit for subscriptions any more than they would make allowances for their patients failing to promptly meet their bills for professional services. Selah!

